# Whole-Genome Sequencing for National Surveillance of *Shigella flexneri*

**DOI:** 10.3389/fmicb.2017.01700

**Published:** 2017-09-19

**Authors:** Marie A. Chattaway, David R. Greig, Amy Gentle, Hassan B. Hartman, Timothy J. Dallman, Claire Jenkins

**Affiliations:** Gastrointestinal Bacteria Reference Unit, Public Health England London, United Kingdom

**Keywords:** *Shigella flexneri*, whole-genome sequencing, surveillance, outbreaks, phylogeny

## Abstract

National surveillance of *Shigella flexneri* ensures the rapid detection of outbreaks to facilitate public health investigation and intervention strategies. In this study, we used whole-genome sequencing (WGS) to type *S. flexneri* in order to detect linked cases and support epidemiological investigations. We prospectively analyzed 330 isolates of *S. flexneri* received at the Gastrointestinal Bacteria Reference Unit at Public Health England between August 2015 and January 2016. Traditional phenotypic and WGS sub-typing methods were compared. PCR was carried out on isolates exhibiting phenotypic/genotypic discrepancies with respect to serotype. Phylogenetic relationships between isolates were analyzed by WGS using single nucleotide polymorphism (SNP) typing to facilitate cluster detection. For 306/330 (93%) isolates there was concordance between serotype derived from the genome and phenotypic serology. Discrepant results between the phenotypic and genotypic tests were attributed to novel O-antigen synthesis/modification gene combinations or indels identified in O-antigen synthesis/modification genes rendering them dysfunctional. SNP typing identified 36 clusters of two isolates or more. WGS provided microbiological evidence of epidemiologically linked clusters and detected novel O-antigen synthesis/modification gene combinations associated with two outbreaks. WGS provided reliable and robust data for monitoring trends in the incidence of different serotypes over time. SNP typing can be used to facilitate outbreak investigations in real-time thereby informing surveillance strategies and providing the opportunities for implementing timely public health interventions.

## Introduction

Shigellosis is caused by four species of *Shigella*, including *S. boydii, S. dysenteriae, S. flexneri* and *S. sonnei*, transmitted via the fecal oral route. Symptoms typically start 1–2 days after exposure and include diarrhea, bloody diarrhea abdominal pain, fever, and tenesmus. The burden of shigellosis is highest in developing countries with up to 167 million episodes of diarrhea annually, leading to over a million deaths (Kotloff et al., 1999). A multicenter study of shigellosis in six Asian countries indicated the incidence rate to be highest in children under the age of 4 years old and in adults over 70 years old (von Seidlein et al., 2006). In the United Kingdom, *S. flexneri* is most commonly associated with causing travelers’ diarrhea and outbreaks of gastrointestinal symptoms in men who have sex with men (MSM) (Simms et al., 2015). Furthermore, there are reports of increased intercontinental dissemination of multidrug resistant *S. flexneri* (Baker et al., 2015).

Between 2004 and 2015, 18,266 *Shigella* cases were reported by GBRU for England and Wales with *S. flexneri* accounting for 7075 (39%) of these infections (*S. sonnei n* = 8897, 49%; *S. boydii n* = 1364, 7%; *S. dysenteriae n* = 808, 4%; *Shigella* species unknown *n* = 122, 1%).

*Shigella flexneri* are traditionally serotyped phenotypically using antisera raised in rabbits, although molecular PCR methods have been implemented in a number of reference laboratories (Zhang et al., 2012; Gentle et al., 2016). Serotyping provides limited resolution with serotypes 2a (*n* = 2448, 35%), 3a (*n* = 1476, 21%), 6 (*n* = 1047, 15%), and 1b (*n* = 711, 10%) accounting for 81% of *S. flexneri* cases. Without a higher level of discrimination, outbreak detection is dependent on the identification of epidemiological links between cases belonging to the same serotype.

Whole-genome sequencing (WGS) has been shown to have potential in replacing traditional phenotypic and PCR methods (Gentle et al., 2016) for routine surveillance. This approach has the added value of further discriminating strains by their genetic relatedness to a single nucleotide polymorphism (SNP) level and has been used to investigate multiple gastrointestinal outbreaks at Public Health England (PHE) (McDonnell et al., 2013; Dallman et al., 2015, 2016).

The aim of this study was to compare traditional serotyping with WGS for serotyping *S. flexneri* for routine public health surveillance and to evaluate utility of WGS data to support epidemiologically linked clusters and outbreak investigations.

## Materials and Methods

### Bacterial Strains

Bacterial isolates of *S. flexneri* from 330 cases were submitted to the Gastrointestinal Bacterial Reference Unit between August 2015 and January 2016, from local and regional hospital laboratories in England and Wales. This strain set comprised the following phenotypic serotypes (numbers of isolates belonging to each serotype in parenthesis): 1a (3), 1b (16), 1c (25), 2a (185), 2b (16), 3a (41), 3b (7), 4av/E1037 (9), 6 (19) X (4), Y (4), X and Y (1). Epidemiological data on age, sex, and region of residence were available from laboratory report forms. Travel history was available for 176 of cases. All isolates were serotyped and WGS.

### Serotyping and PCR

Phenotypic identification of *S. flexneri* isolates were confirmed using the Omnilog GenIII MicroPlate (Biolog, Hayward, CA, United States). Serotyping was carried out using standard methods by slide agglutination using both commercially available monovalent antisera (Denka Seiken, Japan) and monoclonal antibody reagents (Reagensia AB, Sweden) and in-house antisera raised in rabbits (Gross and Rowe, 1985). Molecular PCR was carried out on the discrepancies between phenotypic and WGS typing results as previously described (Gentle et al., 2016) and a serotype assigned according to the gene combination detected (**Supplementary Table S1**).

### Whole-Genome Sequencing

Genome sequencing and sequencing analysis were carried out as previously described (Dallman et al., 2015). Genomic DNA extracted using the QiaSymphony DNA extraction platform (Qiagen) from 330 *S. flexneri* was fragmented and tagged for multiplexing with Nextera XT DNA Sample Preparation Kits (Illumina) and sequenced using the Illumina HiSeq 2500 at PHE. A reference database containing the gene sequences encoding the 12 O-antigen synthesis or modification genes described by Sun et al. (2011, 2012a,b), including *wzxc1-5, wzxe1-5, wzx6, gtrI, gtrII, gtrIV, gtrV, gtrX, gtr1c, oac oac1b* and *opt*, was constructed. Using the *GeneFinder* tool (Doumith, unpublished), FASTQ reads were mapped to the *S. flexneri* O-antigen synthesis or modification genes using Bowtie 2 (Langmead and Salzberg, 2012) and the best match to each target was reported with metrics including coverage, depth, mixture and nucleotide similarity in XML format for quality assessment. Only *in silico* predictions of serotype that matched to a gene determinant at >80% nucleotide identity over >80% target gene length were accepted. FASTQ sequences were deposited in the National Center for Biotechnology Information Short Read Archive under the bioproject PRJNA315192 (see **Supplementary Table S2** for SRA identifiers).

### Cluster Detection

Short reads were quality trimmed and mapped to the reference *S. flexneri* serotype 2a strain 2457T (AE014073.1) (Wei et al., 2003) or the reference strain NC_007613 if *S. flexneri* serotype 6, using BWA v0.75 (Li et al., 2009; Bolger et al., 2014). The Sequence Alignment Map output from BWA was sorted and indexed to produce a Binary Alignment Map (BAM) using Samtools (Li and Durbin, 2010). GATK v2.6.5 was used to create a Variant Call Format (VCF) file from each of the BAMs, which were further parsed to extract only SNP positions which were of high quality (MQ > 30, DP > 10, GQ > 30, Variant Ratio > 0.9) (McKenna et al., 2010). Gubbins v2.0.0 (Croucher et al., 2015) was used to identify recombinant regions of the genome which were subsequently masked for phylogenetic analysis. Pseudosequences of polymorphic positions were used to create maximum likelihood trees using RAxML v8.1.17 (Stamatakis, 2014). *De novo* assembly was carried out using Spades 3.5.0 using ‘–careful’ and ‘ -k 21,33,55,65,77,83,91’ options (Bankevich et al., 2012).

To proactively detect outbreaks from WGS data, SNP typing was carried out on *S. flexneri* isolates belonging to clonal complex (CC) 245 and CC145. CC145 mostly comprises *S. boydii* serotypes but includes *S. flexneri* serotype 6, as this *S. flexneri* serotype was misidentified historically (Wirth et al., 2006; Chattaway et al., 2017). Hierarchical single linkage clustering was performed at seven descending thresholds of SNP distance (Δ250, Δ100, Δ50, Δ25, Δ10, Δ5, Δ0) as previously described (Dallman et al., 2016). This clustering results in a discrete seven digit code where each number represents the cluster membership at each descending SNP distance threshold. The resultant SNP addresses describes an isolates position in the *S. flexneri* population structure where two isolates with the same SNP addresses have 0 SNP differences between them.

## Results

### Demographic of Patients

There were 330 isolates reported between 1st August 2015 and 18th January 2016, 236 (71%) were from males, 85 (26%) from females, and for nine (3%) cases the sex was not stated. Travel history was not provided for 154 (47%) cases, 85 cases reported travel 7 days prior to onset of symptoms and 91 (27%) reported that they did not travel during the 7 days prior to onset of symptoms. The most frequently reported destinations were India (*n* = 16, 5%) and Pakistan (*n* = 11, 3%) with other counties accounting for less < 1% each.

### Comparison of WGS Predicted Serotype versus PCR Predicted Serotype Results

Of the 330 cultures tested prospectively by WGS, 306 (93%) had concordant results with phenotypic serotyping. Three of the mismatched results between the phenotypic and genotypic tests were attributed to mutations identified in O-antigen synthesis or modification genes (**Table 1**), including nonsense mutations resulting in early stop codons (*n* = 2) and a frameshift mutation. Repeat testing of sample 4 revealed the discrepancy was due to an auto-agglutination reaction of the strain with the sera (**Table 1**).

**Table 1:** Summary of mismatched phenotypic and genotypic results.

Reference no.	Sample no.	Phenotypic serotype	PCR serotype	WGS serotype	Explanation of mismatch
SRR4787737	1	1a	1b	1b	*OacIb* contains an early stop codon at position 128, resulting in the 1a phenotype
SRR4786841	2	X variant/Y variant	3a	3a	*Oac* contains an early stop codon at position 20 resulting in the X phenotype
SRR5018321	3	Y variant	2a	2a	Frameshift mutation (insertion of two A’s) at position 1116 rendering *gtrII* non-functional and resulting in the Y phenotype
SRR5017471	4	X variant	Y	Y	Auto agglutination strain resulting in incorrect phenotype
SRR4788187 SRR4897598	5–6	1c	1c	1a	No explanation for the mismatch could be determined
Cluster 2 (13 isolates)	7–19	Negative serology	1cv	1cv	Novel serotype
Cluster 4 (five isolates)	20–24	3a	3av	3av	Novel serotype

*Clusters 2 and 4 contain multiple isolates, please see **Supplementary Table S2** for the Reference numbers.*

The remaining mismatches were novel serotype gene profiles detected by WGS, both associated with outbreaks (Cluster 2, *n* = 13; Cluster 4, *n* = 5) (**Tables 1, 2**). Cluster 2 comprised 13 isolates associated with a local community outbreak. The isolates failed to agglutinate any of the serotype specific *S. flexneri* antisera and were positive for *wzx1-5* and *gtrIc* gene targets in the PCR but were negative for *gtrI*. Typical strains of serotype 1c are positive for *wzx1, gtrI*, and *gtrIc* (**Supplementary Table S1**). These strains were designated 1c variants (1cv). Cluster 4 comprised five isolates of *S. flexneri* associated with an outbreak of gastrointestinal symptoms in five captive chimpanzees phenotypically identified as serotype 3a. The isolates had *wzx1-5, oac*, and *grtX* but were also positive for *gtrII* and were designated 3a variant (3av).

**Table 2:** Summary of Clusters detected by WGS.

Cluster no.	No. cases	Travel	M:F ratio	Age range	Serotype	Epidemiological context/transmission route	SNP address	Minimum SNP difference	Maximum SNP difference	Median SNP difference
1	121	7	119:0^∗^	20–63	2a	MSM	34.42.42.42.#	0	40ˆ	17
2	13	0	4:9	23–96	$1cv	Community	3.45.45.46.46.46.47	0	0	0
3	7	1	3:4	21–53	2b	Restaurant	8.9.9.138.140.154.167	0	0	0
4	5	N/A	N/A	N/A	$3av	Captive chimpanzees	45.130.197.328.353.405.#	1	4	2.5

*Table summarising the four clusters (containing five or more isolates) detected between 1st August 2015 and 18th January 2016. SNP address: hierarchical single linkage clustering performed on the pairwise SNP difference between all isolates at descending distance thresholds (250, 100, 50, 25, 10, 5, 0). Each number of the SNP address represents the cluster membership at that threshold level. SNP addresses with the ‘#’ suffix match all subsequent clusters within that SNP address. ˆ SNP distance corrected for recombination. ^∗^2 cases gender were unknown and not included the ratio. $ determined as variant (v) due to absence of gtrl for 1cv or additional presence of gtrll for 3av.*

### Outbreak Investigation Using SNP Clustering Typing

Analysis of the WGS data organized 161 of the 330 isolates into 36 five SNP single-linkage clusters of two or more isolates associated with CC245 and one cluster in CC145. The median number of cases in these clusters was 2 and ranged from 2 to 27; 32 (89%) clusters investigated comprised less than 5 isolates (**Table 2**). It was not possible to identify epidemiological links associated with these small clusters using only the limited epidemiological data available from laboratory report forms. However, 9/32 (28%) comprised at least one case reporting recent travel abroad prior to onset of symptoms.

During the period of the study, four outbreaks were identified following routine surveillance of local hospital reports of gastrointestinal symptoms caused by *S. flexneri*. SNP typing confirmed that all the isolates belonging to each outbreak cluster were closely related and monophyletic. Cluster 1 was the largest cluster and had a high male to female ratio with the 97% of cases being adult males (for two cases the gender was not stated) (**Table 2**). Isolates from this cluster were observed throughout the study period. The minimum SNP distance between isolates in this cluster was zero with the median distance 17. The maximum SNP distance between any two isolates was 64, however the majority of these SNPs were caused by a transposase mediated recombination of *pic*, encoding a serine protease. This demographic of adult males has previously been shown to be characteristic of clusters linked to sexual transmission among the MSM community (Borg et al., 2012; Gilbart et al., 2015; Baker et al., 2015; Simms et al., 2015). Cluster 1 was part of larger outbreak of *S. flexneri* serotype 2a previously described by Simms et al. (2015).

Clusters 2 and 3 were community outbreaks and cases were geographically linked. They were temporally restricted and genetically homogenous, exhibiting 0–1 SNPs difference in the core genome between isolates. Despite a thorough epidemiological investigation, the source and route of transmission associated with Cluster 2 could not be determined. Cluster 3 was linked to consumption of contaminated food at a restaurant, most likely due to an infected food handler. Cluster 4 was associated with an outbreak of gastrointestinal symptoms in a group of captive chimpanzees with a minimum SNP distance between isolates of one and a maximum SNP distance of four.

**FIGURE 1:**
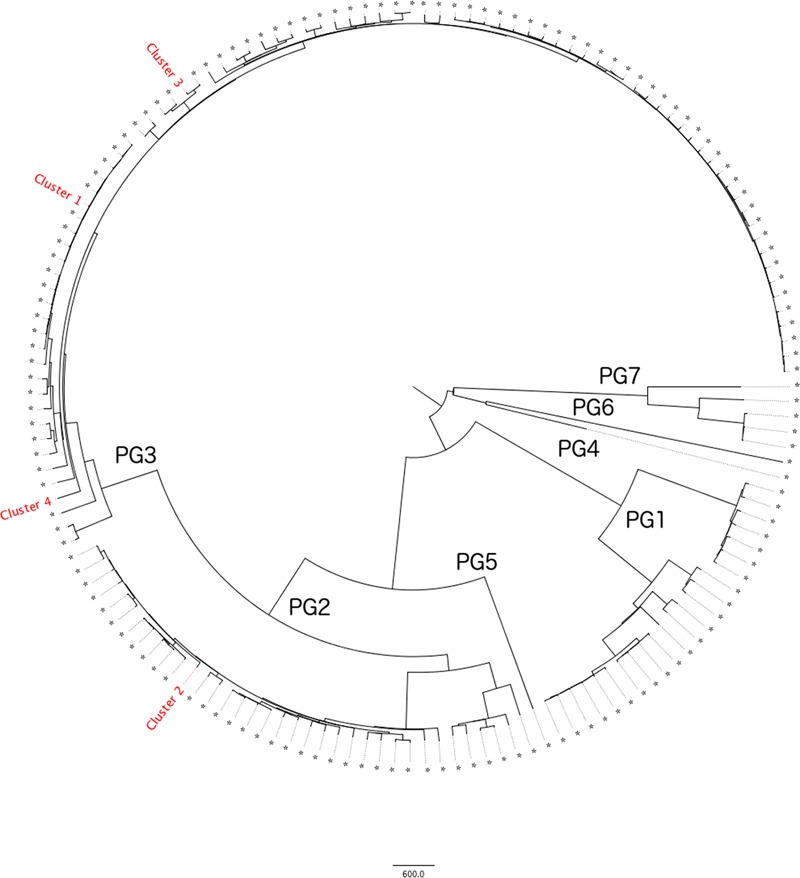
Maximum Likelihood tree showing a single representative from each 10 SNP single linkage cluster (*N* = 156) within the PHE clonal complex 245 database (*N* = 1333). Phylogenetic Groups as defined by Connor et al. (2015) are listed 1–7 and the four outbreak clusters annotated. Refer to **Table 2** for details of the outbreak clusters.

The *S. flexneri* population structure clusters into seven distinct phylogenetic groups (PGs) (Connor et al., 2015). Phylogenetic analysis of the diversity of CC245 in the PHE collection is shown in **Figure 1**. Four out of seven clades are represented by samples received by PHE through routine surveillance. With respect to the clusters described in this study, Clusters 1, 3, and 4 fall within PG3 and Cluster 2 falls within PG1.

## Discussion

This study showed WGS to be a robust and reliable method for serotyping *S. flexneri* isolates and provided additional strain discrimination at the SNP level. There was high correlation between phenotypic serotyping and WGS serotyping (93%) thus facilitating the prospective comparison of WGS data with historical phenotypic data and ensuring continuity for monitoring trends in the incidence of different serotypes over time.

In three of the mismatches, the WGS derived serotype was shown to predict serotype based on gene presence which may not be expressed phenotypically. WGS provided insight on the effect of mutations on O-antigen modification genes and potential mechanisms that inactivate phenotypic expression. WGS analysis also identified novel serotype gene profiles associated with two outbreaks.

Serotype is not a robust phylogenetic marker, as the O-antigen synthesis/modification genes are encoded on mobile genetic elements (prophages) (Connor et al., 2015). Prior to the implementation of WGS, detection of outbreaks of *S. flexneri* at PHE relied on the identification of epidemiological links between cases as serotyping was not discriminatory enough to detect outbreaks during routine surveillance. During this study, SNP typing provided microbiological evidence that the isolates associated with each of the four outbreaks identified were closely related. SNP typing has been used previously to investigate outbreaks of *S. sonnei*, and this study provides further evidence of the utility of this approach (McDonnell et al., 2013; Dallman et al., 2016; Mook et al., 2016; Baker et al., 2017).

Three of the outbreak clusters were temporally and genetically restricted with less than five SNP differences observed between outbreak isolates. In contrast, an outbreak representing on-going person-to-person transmission within the MSM community exhibited between zero and 64 SNP differences between isolates. *S. flexneri* may become endemic in defined populations or communities, such as religious communities or sexual networks. Over time circulating strains will accumulate mutations leading to an increase in observed SNPs between cases in that network. Similarly, extended transmission provides greater opportunities for horizontal gene transfer in the population and therefore bioinformatic analyses have to be robust to such influxes of variation.

This heterogeneity in genetic conservation between epidemiologically linked cases highlights the need to be flexible with respect to the case cluster definition. Epidemiological data associated with each outbreak, and WGS analyses of the deeper phylogenetic relationship between isolates, should be used in concert to inform outbreak investigations.

The use of WGS for routine surveillance of *S. flexneri* provides reliable and robust data that can be used to monitor trends in the incidence of different serotypes over time. WGS derived serotyping data ensures backward compatibility with historical phenotypic serotyping data. SNP typing can be used to facilitate outbreak investigations in real-time thereby enhancing surveillance strategies and providing the opportunities for implementing rapid public health interventions.

## Informed Consent

Informed consent was not required as all data in this study was anonymized.

## Author Contributions

DG and AG performed the DNA extractions, PCR, phenotypic serotyping and identification. MC and CJ implemented the wet lab WGS pipelines and performed analysis. TD, HH, and DG performed bioinformatic analysis. MC and CJ wrote the manuscript and TD, DG, and HH contributed to the manuscript.

## Conflict of Interest Statement

The authors declare that the research was conducted in the absence of any commercial or financial relationships that could be construed as a potential conflict of interest.

## Supplementary Material

TABLE S1 Serotype by gene presence.

TABLE S2 Sample list and results.
