# Supplementary material for: Whole-Genome Sequencing for National Surveillance of Shigella flexneri
Source: Front Microbiol. 2017 Sep 19;8:1700. doi: 10.3389/fmicb.2017.01700 (PMC5610704; doi:10.3389/fmicb.2017.01700)
Supplement: TABLE S1 — Serotype by gene presence. [file Table_1.docx]

### Supplementary Table 1: Serotype by gene presence

| **Serotype** | **wzx 1-5** | **wzx 6** | **oac** | **oacIb** | **opt** | **gtrI** | **gtrIc** | **gtrII** | **gtrIV** | **gtrV** | **gtrX** | **REF** |
| --- | --- | --- | --- | --- | --- | --- | --- | --- | --- | --- | --- | --- |
| 1a | + | - | - | - | - | + | - | - | - | - | - | [1] |
| 1b | + | - | + | - | - | + | - | - | - | - | - | [1] |
| 1bv | + | - | - | + | - | + | - | - | - | - | - | [2] |
| 2a | + | - | - | - | - | - | - | + | - | - | - | [1] |
| 2b | + | - | - | - | - | - | - | + | - | - | + | [1] |
| 3a | + | - | + | - | - | - | - | - | - | - | + | [1] |
| 3av | + | - | + | - | - | - | - | + | - | - | + | This study |
| 3b | + | - | + | - | - | - | - | - | - | - | - | [1] |
| 4a | + | - | - | - | - | - | - | - | + | - | - | [1] |
| 4av  (E1037) | + | - | - | - | + | - | - | - | + | - | - | [3] |
| 4b | + | - | + | - | - | - | - | - | + | - | - | [1] |
| 5a | + | - | (+) | - | - | - | - | - | - | + | - | [1, 4] |
| 5b | + | - | (+) | - | - | - | - | - | - | + | + | [1, 4] |
| 6 | - | + | - | - | - | - | - | - | - | - | - | [1] |
| 7a (1c) | + | - | - | - | - | + | + | - | - | - | - | [1] |
| 1cv | + | - | - | - | - | - | + | - | - | - | - | This study |
| 7b | + | - | + | - | - | + | + | - | - | - | - | [1] |
| X | + | - | - | - | - | - | - | - | - | - | + | [1] |
| Xv | + | - | - | - | + | - | - | - | - | - | + | [3] |
| Y | + | - | - | - | - | - | - | - | - | - | - | [1] |
| Yv | + | - | - | - | + | - | - | - | - | - | - | [3] |

1. Sun Q, Lan R, Wang Y, Zhao A, Zhang S, Wang J, et al. Development of a multiplex PCR assay targeting O-antigen modification genes for molecular serotyping of Shigella flexneri. J Clin Microbiol. 2011;49(11):3766-70. doi: 10.1128/JCM.01259-11. PubMed PMID: 21880974; PubMed Central PMCID: PMCPMC3209073.

2. Sun Q, Lan R, Wang Y, Wang J, Xia S, Wang Y, et al. Identification of a divergent O-acetyltransferase gene oac 1b from Shigella flexneri serotype 1b strains. Emerg Microbes Infect. 2012;1(9):e21. doi: 10.1038/emi.2012.22. PubMed PMID: 26038428; PubMed Central PMCID: PMCPMC3630932.

3. Sun Q, Knirel YA, Lan R, Wang J, Senchenkova SN, Shashkov AS, et al. Dissemination and serotype modification potential of pSFxv_2, an O-antigen PEtN modification plasmid in Shigella flexneri. Glycobiology. 2014;24(3):305-13. doi: 10.1093/glycob/cwt115. PubMed PMID: 24379081.

4. Ashton PM, Baker KS, Gentle A, Wooldridge DJ, Thomson NR, Dallman TJ, et al. Draft genome sequences of the type strains of Shigella flexneri held at Public Health England: comparison of classical phenotypic and novel molecular assays with whole genome sequence. Gut Pathog. 2014;6(1):7. doi: 10.1186/1757-4749-6-7. PubMed PMID: 24684748; PubMed Central PMCID: PMCPMC3972513.
